# Supplementary figures and images for: Long non-coding RNA, LINC01614 as a potential biomarker for prognostic prediction in breast cancer
Source: PeerJ. 2019 Nov 14;7:e7976. doi: 10.7717/peerj.7976 (PMC6858983; doi:10.7717/peerj.7976)

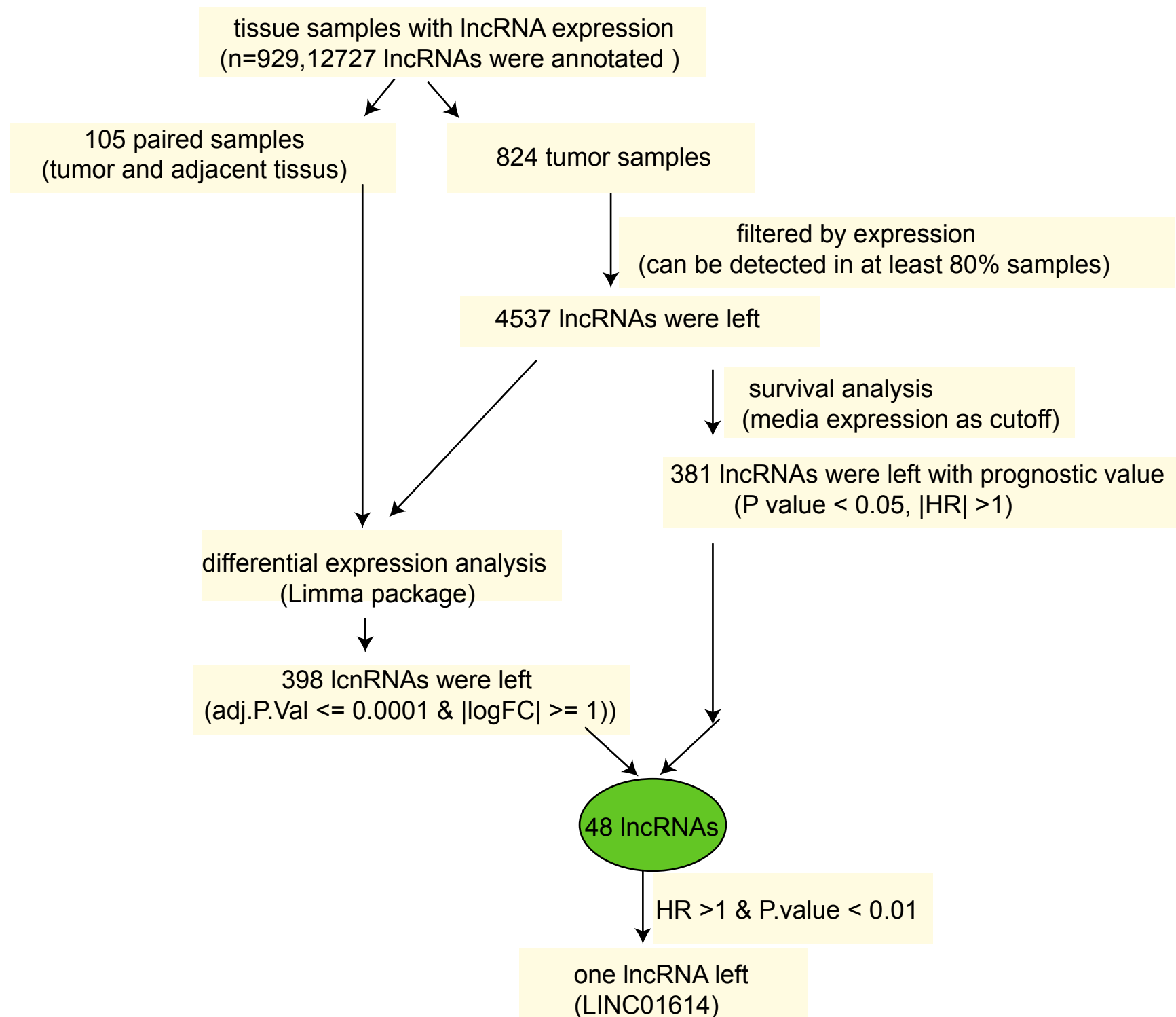

Supplement: Supplemental Information 1 [file peerj-07-7976-s001.pdf]

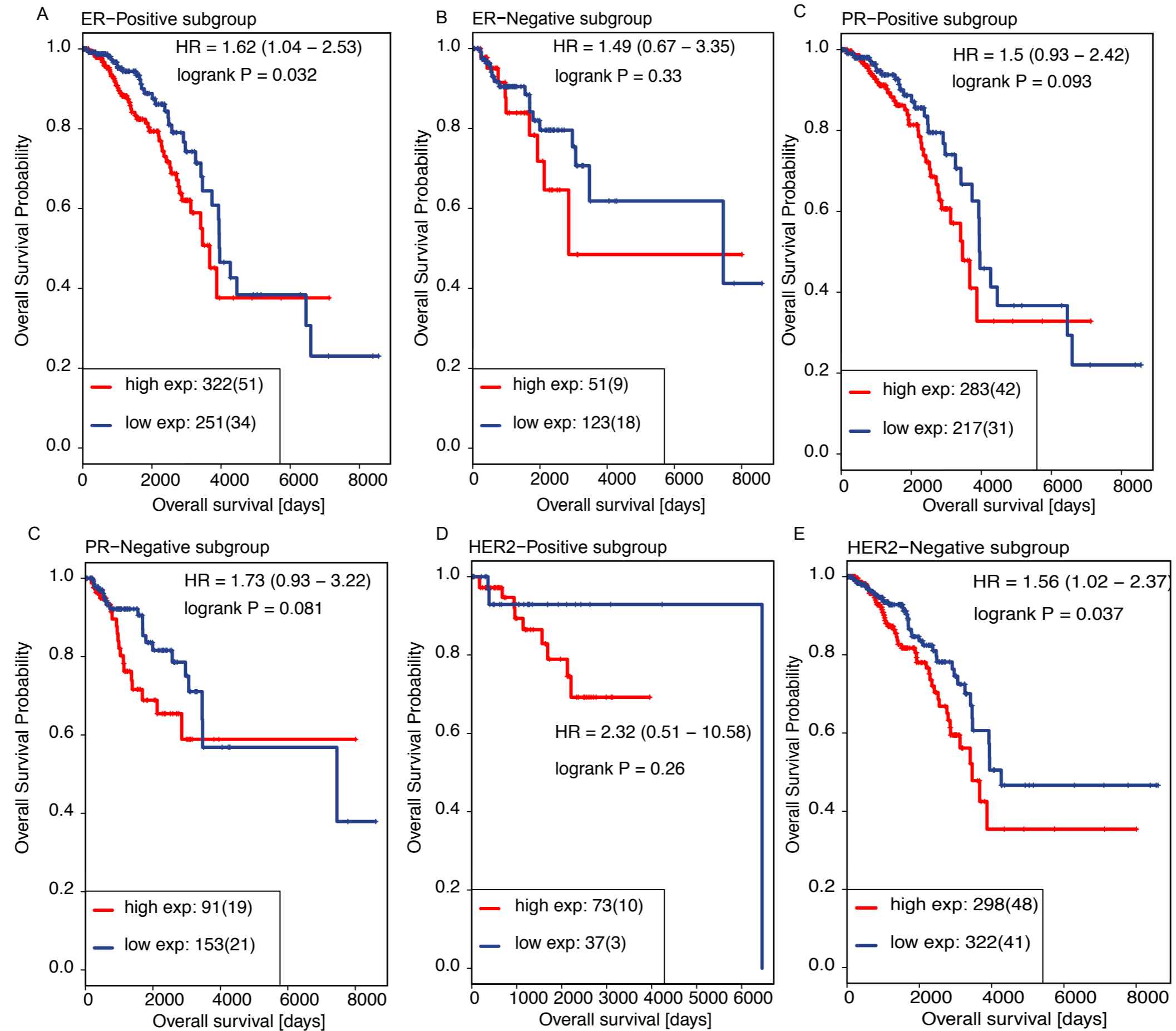

Supplement: Supplemental Information 2 — (A) Kaplan−Meier estimates of OS in ER+ samples (n = 573). (B) Kaplan−Meier estimates of OS in ER- samples (n = 174). (C) Kaplan−Meier estimates of OS in PR+ samples (n = 500). (D) Kaplan−Meier estimates of OS in PR+ samples (n = 244). (E) Kaplan−Meier estimates of OS in HER2+ samples (n = 110). (F) Kaplan−Meier estimates of OS in HER2+ samples (n = 620). The tick marks on the Kaplan−Meier curves indicated the censored cases. The differences between the two curves were determined by the two-side log-rank test. [file peerj-07-7976-s002.pdf]

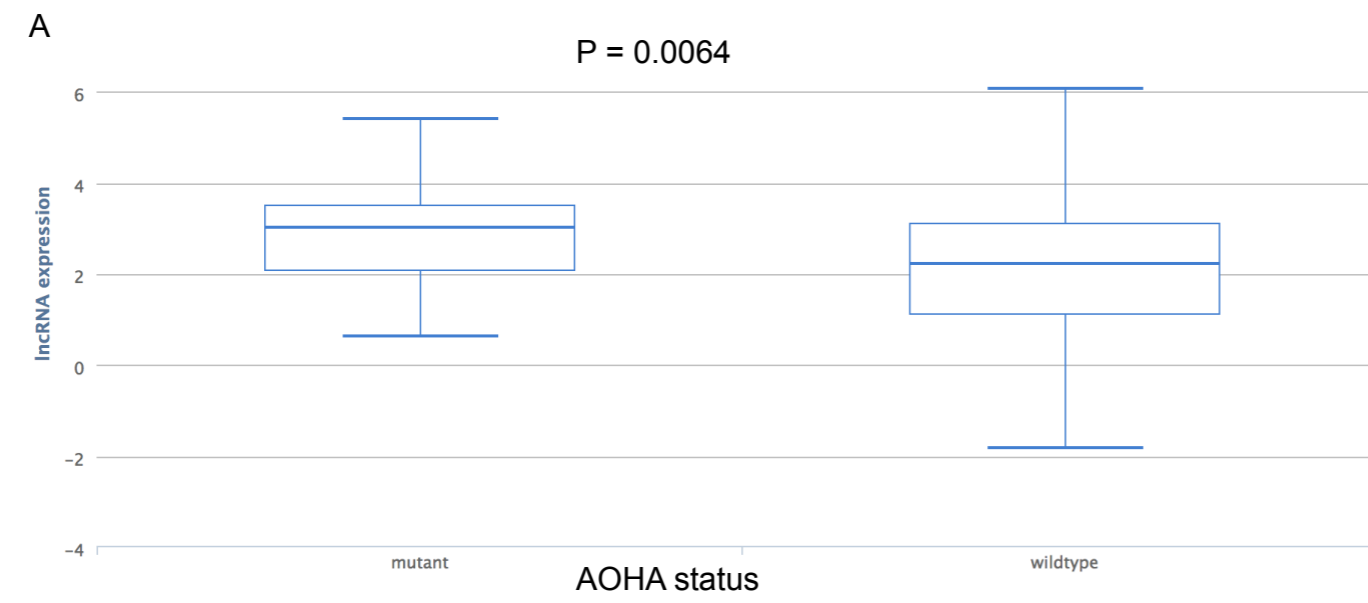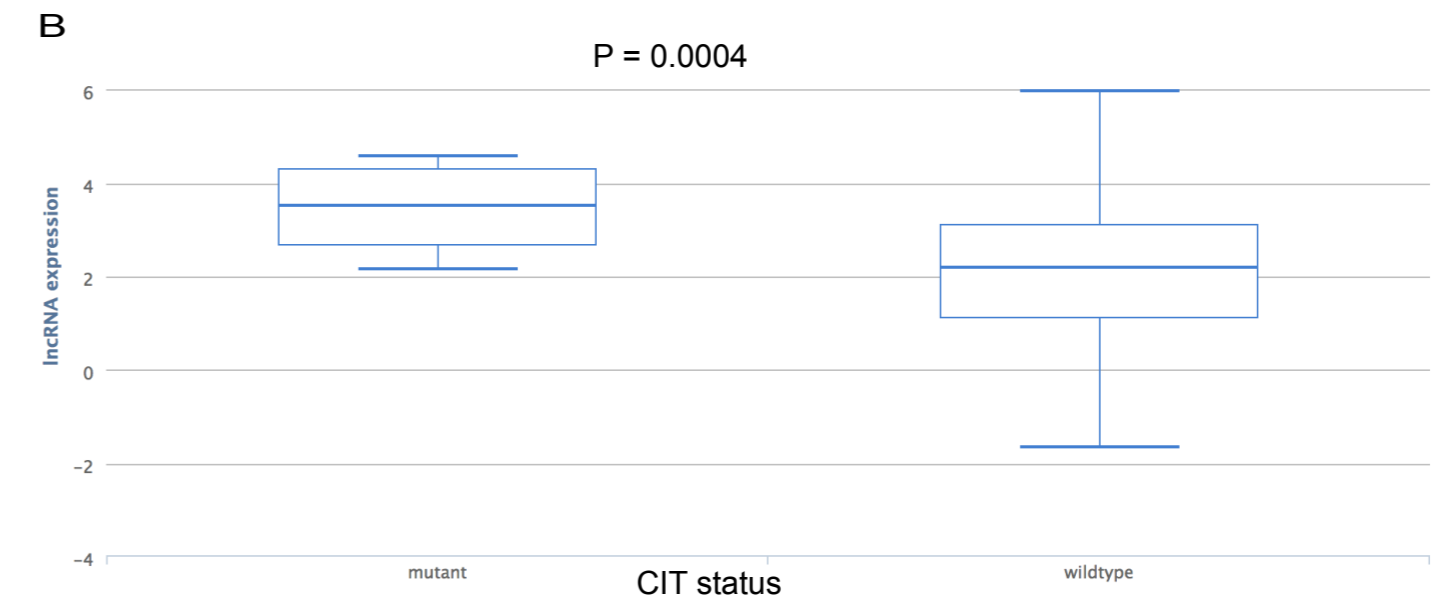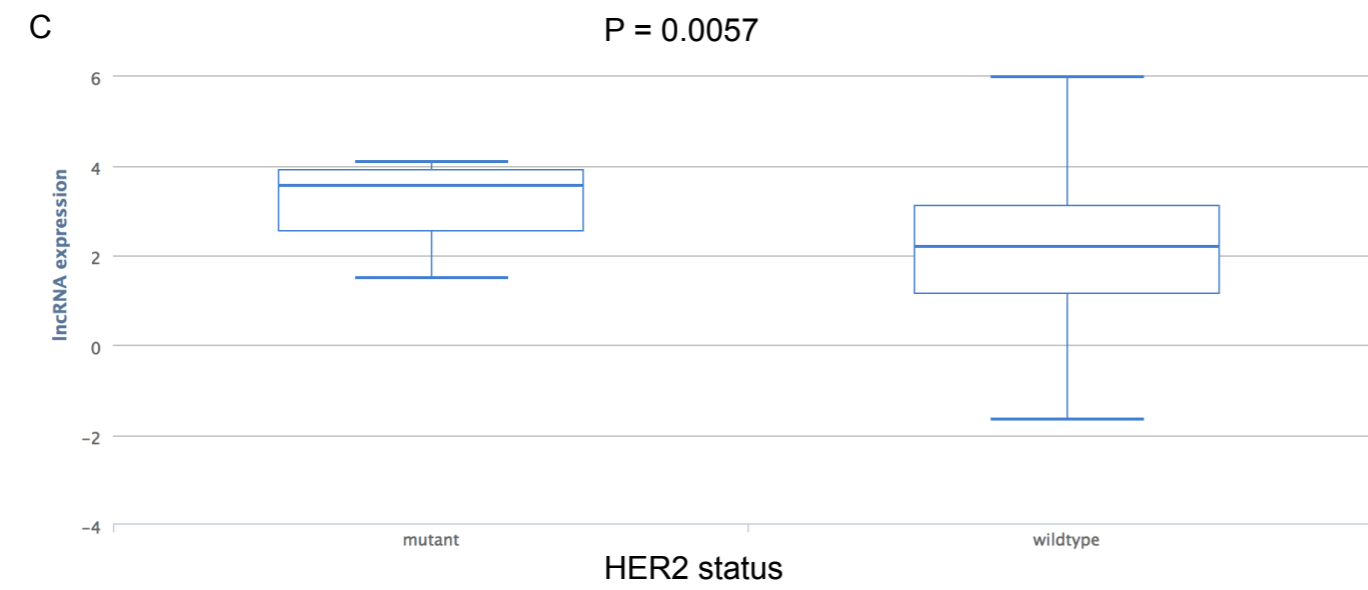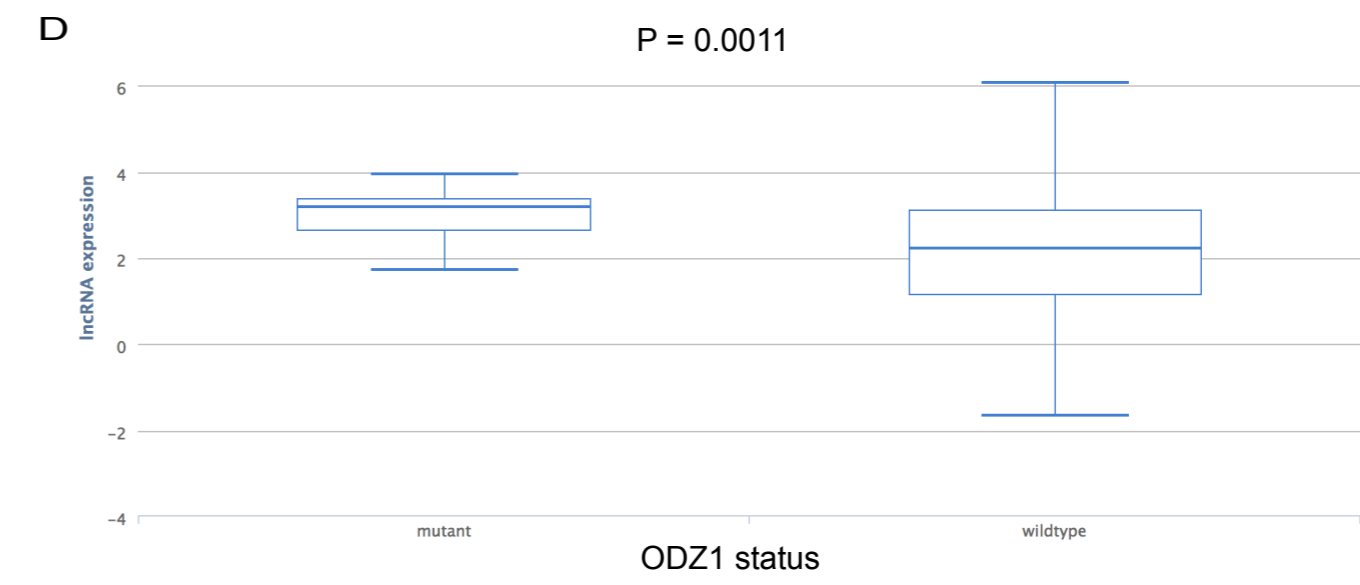

Supplement: Supplemental Information 3 — (A) AOHA (mutant vs. wildtype). (B) CIT (mutant vs. wildtype). (D) HER2 (mutant vs. wildtype). (E) ODZ1 (mutant vs. wildtype). [file peerj-07-7976-s003.pdf]
